# Emergency contraceptive knowledge, utilization and associated factors among secondary school students in Wolkite town, southern Ethiopia, cross sectional study

**DOI:** 10.1186/s40834-020-00119-4

**Published:** 2020-09-15

**Authors:** Dereje Mesfin

**Affiliations:** Department of Public Health, Wolkite University College of Medicine and Health Science, Wolkite, Ethiopia

**Keywords:** Emergency contraceptive, Knowledge, Practice, Secondary schools

## Abstract

**Background:**

Ethiopia is one of the sub-Saharan African countries with high maternal mortality and morbidity, unsafe abortion and adolescent births. Despite different policy measures taken by the government to improve sexual and reproductive health among adolescents their success is not well studied in Ethiopia. The objective of this study is to explore emergency contraceptive related knowledge, practice and its determinants among secondary school students in southern Ethiopia.

**Methods:**

An institution-based cross-sectional study was conducted in selected high schools of Wolkite town, Southern Ethiopia from December to November 2019. Single population proportion formula was used to calculate sample size. A total of 327 female students participated in the study with a total response rate of 97%. Data were collected using a self-administered, structured questionnaire and cleaned, entered and analyzed using Statistical package for social science software version 21.

**Result:**

153 (54.8%) of the study participants had good knowledge about emergency contraceptives and only (40.5%) of sexually active participates used emergency contraceptives after unprotected sex. Type of admission and grade level of participants and discussion of reproductive health related issues with parents were significantly associated with good knowledge of Emergency contraceptive. Having partner and grade level of students were among the significant determinants of emergency contraceptive utilization.

**Conclusion:**

The study showed an acceptable level of emergency contraceptive knowledge but only less than half of sexually active respondents used emergency contraceptives. To prevent unintended pregnancy among secondary school students sexual and reproductive health education should be given to students starting from their enrollment. Furthermore, parents should be encouraged to freely discuss sexual and reproductive health matters with their children.

## Background

Emergency contraceptives (EC) are modern, safe, therapeutically efficient and cost-effective methods of contraception which are commonly used after unprotected sex, missing of regular contraception dose, following sexual abuse and nonuse of contraception to prevent unplanned pregnancy [1]. Emergency contraceptives avoid pregnancy by interfering in the physiologic process of fertilization, implantation, and tubal transportation of sperm and ovum [2].

Emergency contraceptives are administered after the unprotected sexual act, unlike other contraceptive methods which are used regularly or before the sexual intercourse [2]. Proper use of emergency contraceptive can reduce the occurrence of unintended pregnancy and risk of an abortion if used before the potential time of implantation, especially within 72 h after unprotected sexual intercourse [3–5].

There are two categories of emergency contraceptives the firs category includes emergency contraceptive pills such as progestin-only pills (POPs) and combined oral contraceptive pills (COCs). The second category includes intrauterine devices (IUCDs), IUCDs known to be therapeutically effective if they are inserted within 7 days of unprotected sexual intercourse [6].

Globally 250 Million pregnancies take s place each year among these pregnancies about 25% of them are unintended and 20% of those mothers with unintended pregnancy undergo induced abortion [7]. According to an estimate of world health organization in Africa nearly 5.5 million women have unsafe abortions each year and about 59% of these unsafe abortions are among young women [8].

Alarmingly more than 60% pregnancies among adolescents in Ethiopia are unwanted and end up with unsafe abortions and most of this pregnancies happened either due to low level of knowledge, poor attitudes or lack accessibility to contraceptive, furthermore findings from previous studies showed that the level of knowledge regarding EC is below 50% and the practice level is below 10% [9–12].

In Ethiopia early initiation of sex is among the major challenges posed on the young generation studies showed that the median age to start sex for women in Ethiopia is 16 years, additionally despite the fact that sizable number of Ethiopians know about modern family planning methods most of the do not practice them [13]. According to the report of Ethiopian demographic health survey (EDHS 2016) in Ethiopia only 36% of reproductive age women have access to contraceptives and from this only 4% of them use emergency contraceptive [14].

Adolescence is the age where most sexual characteristics develop and high school are places where adolescents spend most of their times. Different studies showed that significant proportions of that student in high schools of Ethiopia are experienced sexual intercourse at least once. For instance studies conducted among high school and preparatory students in Mizan and Harar cities showed that few of the students utilized ECs after unprotected sexual intercourse [15, 16].

Several policy measures were taken by the government to avert unacceptably high level of maternal health related problems in Ethiopia, for instance universal access of sexual and reproductive health to adolescent was endorsed as one of the targets in the revised millennium development goal (MDGs) of the country yet the success of such policy measure among adolescents is not well studied in Ethiopia. Consequently no school based appropriate strategies were designed [17].

Most of the studies conducted concerning emergency contraceptives knowledge and practice focused on university and college student and little focus was given for high schools but cultural transformations and globalization effects which resulted in increased adolescent sexual activity and lower age at first sex makes high schools an important focus area to assess emergency contraceptive knowledge and practice [9–12, 18]. Thus, the objective of this study is to assess the level of emergency contraceptive knowledge, practice, and its determinant factors among high school students.

## Methods

### Study area and period

This study was conducted in selected secondary schools of Wolkite town, Southern Ethiopia from December to November 2019. Wolkite town is located 157 km from Addis Ababa capital of Ethiopia. According to the Education office of Wolkite town, there are a total of four high schools in Wolkite town namely, Yaberus Secondary and Preparatory School (YSPS), Ras Zeselassie Secondary School (RZSS) and Wolkite Secondary School (WSS) and Melke Tsedik Secondary School (MTSS). Currently, there are a total of 3527 students who are admitted to those four secondary schools both in regular and night programs and among these 1716 were Males and 1811 of them were females. Two secondary schools YSPS and RZSS were randomly selected for this study.

### Study design

An institution-based cross-sectional quantitative study was conducted in selected Secondary schools of Wolkite town.

### Source populations

All-female students who were attending secondary schools in Wolkite town were the source populations of this study on which inference can be drawn.

### Study populations

Randomly selected female students who were admitted to the selected (YSPS and RZSS) secondary schools of Wolkite town during the study period.

### Eligibility criteria

#### Inclusion criteria

Female students enrolled in YSPS and RZSS in Wolkite town who are willing to participate in the study were included in the study.

### Sample size determination and sampling technique

The minimum required sample size was calculated using a formula for single population proportion considering the following assumptions: 95% confidence interval, 5% of margin of error, 70% prevalence of knowledge of emergency contraceptive, which resulted in a sample size of 369 [19, 20].

Since the size of the source population is below 10,000, the finite population correction formula was employed which brought the sample size to 306, by taking possible non-response rate of 10% then final calculated sample size happened to be 337.

**Fig. 1:**
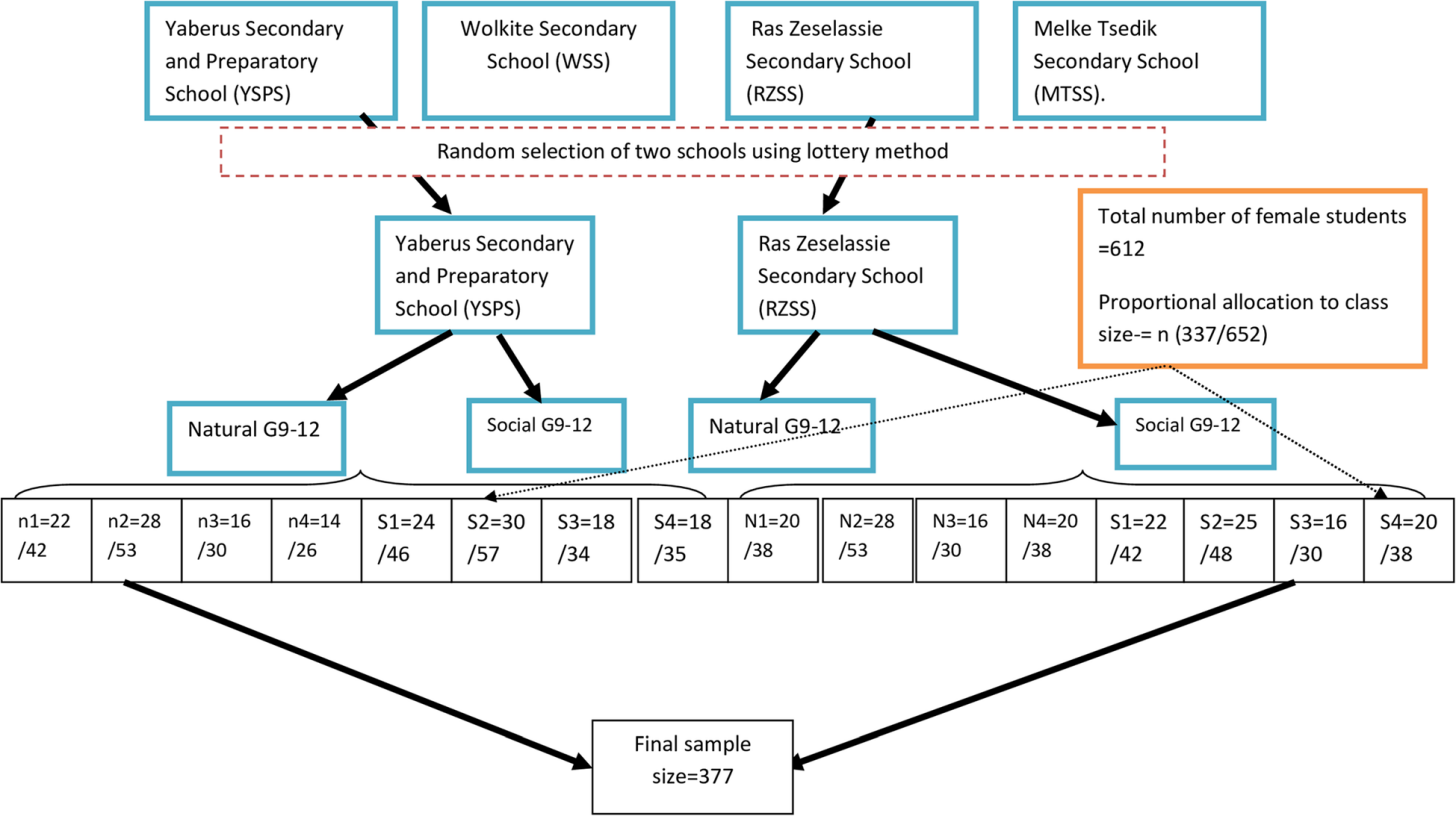
Schematic presentation of the sampling procedure for data collection form secondary schools of Wolkite town southern Ethiopia, 2019

A multi-stage sampling technique was used to select the study participants. First, two schools were randomly selected among the four secondary schools the selected highs schools (YSPS and RZSS) have a total of 612 female students in 60 sections with both in the social and natural science streams each with a different number of female students (42. 53.30, 26, 46, 57, 34, 35, 38 …) as depicted in the Schematic presentation of the sampling procedure for data collection in Fig. 1 then the study units were selected using systematic random sampling technique after proportional allocation of the sample size into each section based on the total number of female students in each section (see Fig. 1).

### Data collection technique

Data were collected using a structured self-administered questionnaire. The instrument was first developed in English and translated into Amharic (local language) and back-translated into English to ensure its consistency. The instrument was developed after reviewing different works of literature on related studies. To ensure instrument quality, pre-test was conducted on 5% (17 female students) of the total sample size in Emdibir Secondary school which is outside of the study area.

After the pre-test all the participants were directly contacted and asked about the clarity questions in the instrument and the data collectors were also asked if there was any kind of difficulty on the data collection process, accordingly, some modifications were made on sequence and wording of questions in the instrument based on their suggestions before the actual data collection process. The questionnaire has different parts which assess Sociodemographic variables, sexual activity, emergency contraceptive knowledge, and practice.

### Data collectors and supervisors

Four data collectors who had a diploma in health-related fields with or without previous experience in data collection but fluent in local and English languages were selected for data collection.

Two supervisors with a BSc degree and previous experience with supervision of data collection were recruited from the nearby health centers to oversee the data collection process.

The data collection program was arranged in collaboration with the school directors and teachers. The questionnaires were distributed and collected back from the study subjects before class started.

### Data processing and analysis

The collected data were checked for completeness and accuracy, cleaned, entered, and analyzed using statistical package for social sciences (SPSS) version 21 software. Different descriptive statistics such as percentages mean and standard deviations were computed for different study variables and presented in charts and tables both binary and multivariate logistic analysis was conducted to determine predictors of Emergency contraceptive knowledge and practice. *P* < 0.05 were used to declare statistical significance.

“Good knowledgeable to EC” refers to a female student who answered correctly and their scores are above or equal to the mean score 4 of the total seven knowledge related questions such as types of EC, correct time to use EC, safety of EC, effectiveness & side effects of EC... (see Table 3); “poor knowledgeable” refers to a female student who correctly answered knowledge related questions and their scores are below the mean score [20].

“Practice of emergency contraceptive” refers that a female student who ever used emergency contraceptive after unprotected sexual intercourse to prevent unintended pregnancy after admission to high school in their lifetime.

## Results

### Socio-demographic characteristics

A total of 327 female students have participated in the study with 97% total response rate. Among this study participants majority 287(87.7%) of them were admitted in a regular (day time) program and 176 (53.8%) of them were followers of orthodox Christianity (see Table 1).

**Table 1:** Sociodemographic characteristics of female study participants in selected secondary schools of Wolkite town, southern Ethiopia, 2019

S.N	Variables	Frequency (*n* = 327)	Percentages
1.	Age category		
	14–18	210	64.22
	19–23	110	33.6
	24–28	17	5.1
	Total	327	100
2.	Admission type		
	Regular	284	86.8
	Night	43	13.1
	Total	327	100
4.	Religion		
	Orthodox	176	53.3
	Muslim	95	29.0
	Adventist	28	8.5
	Protestant	18	5.5
	Others	10	3.0
	Total	327	100
5.	Marital status		
	Married	25	7.6
	Never married	302	92.3
	Total	327	100
6.	Ethnicity of the respondent		
	Gurage	188	57.4
	Oromo	71	21.7
	Amhara	50	15.2
	Kebena	15	4.5
	Others	3	0.9
	Total	327	100

**Fig. 2:**
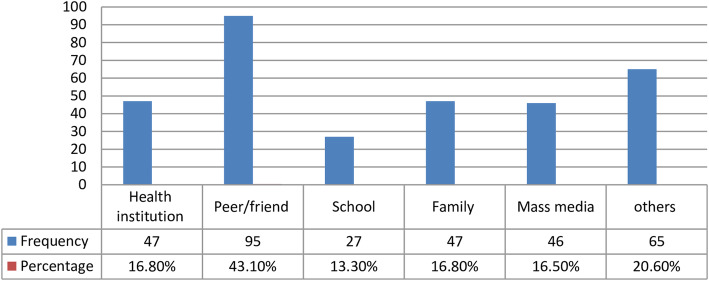
Grade level of respondents in selected secondary schools of Wolkite town, Southern Ethiopia, 2019

From the total study participants, 210(64.22%) of them were within the age group of 14–18 years and the mean age of the participants was 18.08 (SD ±2.91) years. The majority of the students were from grade 10 (see Fig. 2).

### Socio- demographic characteristics of study participants families or care takers

From the total study participants, 281 (85.9%) of them reported that their parents are urban residents and 187 (57.3%) of the students mentioned that they are currently living with both of their parents. Majority 130(39.7%) of the student said that their mothers had primary education, furthermore, most of the students 175 (53.5%) mentioned that their fathers attended secondary education. Concerning discussion on reproductive health issues with their parents 140 (42.8%), the study participants reported that they have discussed those matters with their parents (see Table 2).

**Table 2:** Socio-demographic characteristics of study participant’s families or caretakers selected secondary schools of Wolkite town. Southern Ethiopia, 2019

S.N	Variables	Frequency n(327)	Percentages
1.	Family or caretaker residence		
	Urban	281	85.9
	Rural	46	14.6
	Total	327	100
2.	Person with whom respondents are living		
	Mother alone	44	13.4
	Father alone	27	8.2
	Both parents	187	57.3
	Alone	41	12.5
	With friend	16	4.8
	Others	8	2.4
	Total	327	100
3.	Mothers educational level		
	Illiterate	122	37.8
	Primary education	130	39.7
	Secondary education	58	17.7
	Above secondary education	17	5.1
	Total	327	100
4.	Father educational level		
	Illiterate	24	7.3
	Primary education	82	25.0
	Secondary education	175	53.5
	Above secondary education	46	14.0
	Total	327	100
5.	Discussion about RH issues with parents		
	Yes	140	42.8
	No	187	57.1
	Total	327	100

### Knowledge on emergency contraceptives

**Fig. 3:**
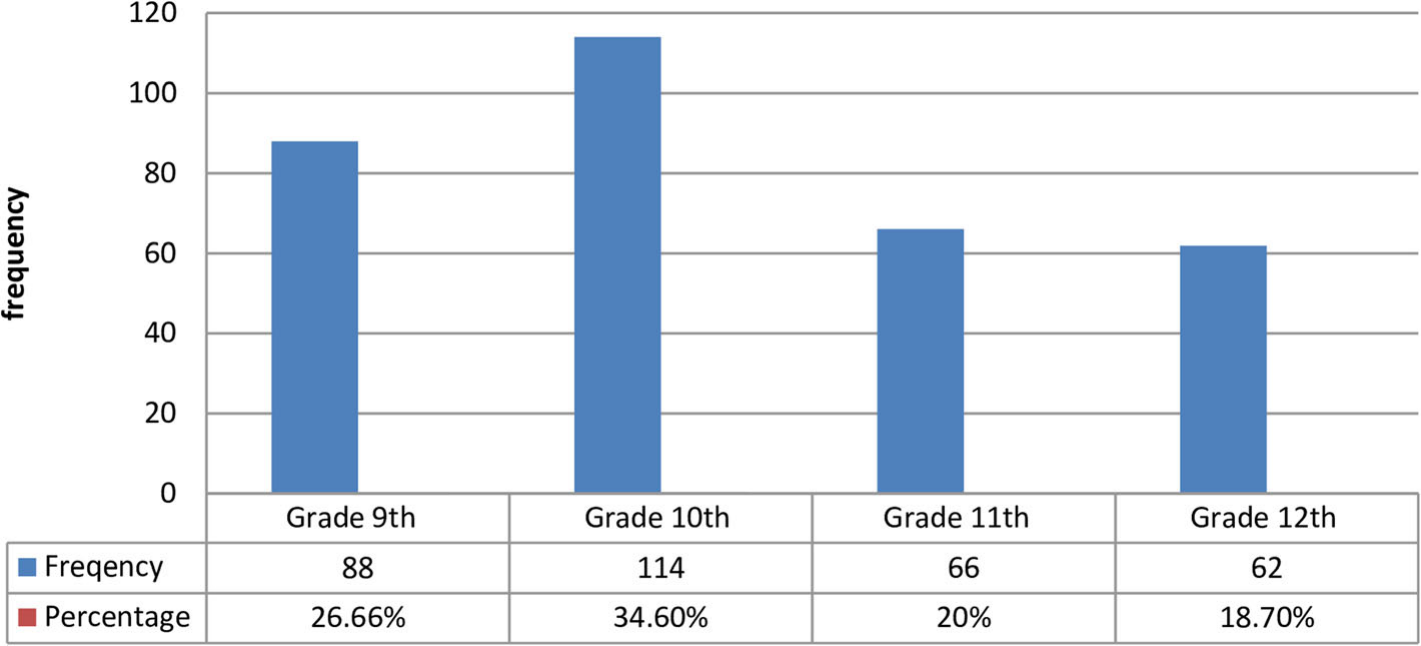
Source of information about emergency contraceptives in among secondary school female students of Wolkite town, Southern Ethiopia, 2019

Overall summary of participant’s level of knowledge regarding EC indicated that from the total study participants about 153 (54.8%) of the study participants had good knowledge about EC. Regarding their source of information majority of the total respondents 95(34.10%) have got the information from their peers which is followed by 47(16.8%) from health institutions family and media (see Fig. 3 and Table 3).

**Table 3:** Emergency contraceptive knowledge among secondary school students in Wolkite town, southern Ethiopia, 2019

S.N	Variables	Frequency n (327)	Percentages
1.	Places to get emergency contraceptives		
	Pharmacy	157	48.1
	Health centers	93	28.4
	Hospitals	48	14.6
	Others	16	4.8
	Don’t know	13	3.9
2.	Types of Emergency contraceptive female students know		
	COC	144	44.0
	Injectables	75	22.9
	POP	19	5.8
	Implant	31	9.4
	IUCD	12	3.6
	>/=2 of above mentioned methods	46	14.06
3.	Correct time to use the first dose of EC pills		
	Within 72 h	178	54.4
	Within 48 h	43	13.1
	Within 120 h	58	17.7
	Don’t know	48	14.6
4.	Reason for use of Emergency contraceptive		
	Unwanted pregnancy	104	31.8
	Condom slipped/breakage	62	18.9
	Missed pill	47	14.3
	In two or more of the above	88	37.1
	Don’t know	26	7.9
5.	Effectiveness of EC to prevent pregnancy if used properly		
	Highly effective	39	11.9
	Moderately Effective	58	17.7
	Effective	39	11.9
	Uncertain	26	7.9
	Not that much effective	54	17.4
	Don’t know	111	33.9
6.	Appropriate Time use IUCD as EC		
	Within 72 h	64	19.5
	Within 120 h	180	55.0
	Don’t know	83	25.3
7.	Safeness of emergency contraceptive use for most women		
	Safe	113	39.4
	Unsafe	72	26.9
	Not sure	94	33.6

### Practice of emergency contraceptives

**Fig. 4:**
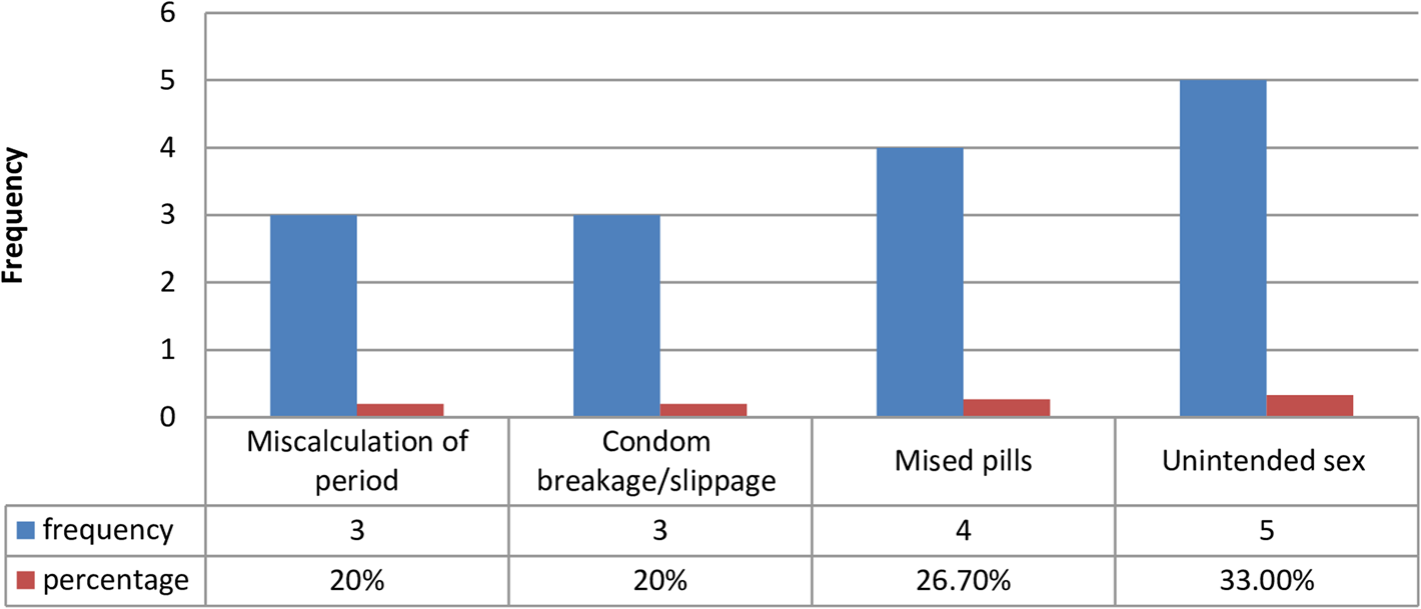
Reasons for the use of EC among female students in secondary schools of Wolkite, Southern Ethiopia, 2019

Out of the total study participants, 90(27.2%) of them had sexual intercourse at least once in their life time, among those of sexually active study participants, 37(41.10%) of them had unprotected sex and of those only 15 (40.5%) of them used EC. All of the respondents who used EC had taken pills and none of them used IUCDs. The main reason for taking EC was unintended sex (33.3%) followed by missing pills 26.7% (see Fig. 4).

### Determinant factors of emergency contraceptive knowledge

Type of admission (AOR = 7.421(1.241–4.041) *P* < 0.026],) and grade level of participants were among the significant predictors of EC knowledge, accordingly female students admitted to the night program and those students senior classes had more knowledge compared to grade nine students (AOR (4.21(3.451–4.172), *P* < 0.0140), AOR = 2.02(1.641–9.071), *P* <  0.035) respectively (see Table 4).

**Table 4:** Factors associated with EC knowledge among secondary school students in Wolkite town, southern Ethiopia, 2019

Variable	Knowledge of EC		COR (95% CI)	AOR (95% CI)	*P*-value
	Yes	No			
Age					
14–18 years	167	43	1	1	0.459
19–23 years	95	3	0.453(0.237–0.507)	1.434(0.101–1.520)	0.234
24–28 years	17	2	0.231(0.045–1.244)	1.50(0.364–4.625)	0.548
Admission type					
Night	41	1	7.025(1.28–12.32)	7.421(1.241–4.041)	0.026*
Regular	238	47	1	1	1
Grade level					
Grade 9th	49	38	1	1	0.021
Grade 10th	106	7	0.34(0.362–0.931)	4.21(3.451–4.172)	0.014*
Grade 11th	66	0	0.251(0.670, 0.975)	0.421(0.521–1.371)	0.872
Grade 12th	58	3	0.543(0.239, 0.439)	2.02(1..641 - 9.071)	0.035*
Father education					
Discussion about RH issues with parents					
Yes	126	14	2.00(1.028, 3.891)	2.721(0.231, − 2.612)	0.013*
No	153	34	1	1	0.412

*CI* Confidence interval, *COR* Crude odds ratio, *AOR* Adjusted odds ratio

*P < 0.05 = indicates statistically significant association

### Factors associated with practice of EC

As it is noted in Table 5, Grade level of students, Grade 11th (AOR (1.812 (0.672–1.278) *P* < 0.038), Grade 12th (AOR 2.83(0.231–1.549), *P* < 0.026), and presence of boyfriend (AOR 5.723 (1.007–1.213), *P* < 0.015) had a statically significant association with the utilization of EC (see Table 5).

**Table 5:** Factors associated with EC utilization among secondary school students in Wolkite town, southern Ethiopia, 2019

Variable	Use of EC		COR (95% CI)	AOR (95% CI)	*P*- Value
	Yes	No			
Admission type					
Regular	5	16	1	1	0.431
Night	10	6	3.813(1.349,4.245)	0.274(0.671–7.281)	0.976
Grade level					
Grade 9th	2	12	1	1	0.021
Grade 10th	8	4	0.343(0.232, 1.458)	0.73 (0.24–7.521)	0.631
Grade 11th	4	5	0.451(0.078,2.528)	1.812(0.672–1.278)	0.038*
Grade 12th	1	1	0.243(0.014, 1.271)	2.83(0.231–1.549)	0.026*
Marital status					
Single	3	17	1	1	0.910
Married	12	4	0.254(0.011, 0.512)	1.24(0.340–3.091)	0.834
Have a boy friend					
Yes	14	9	1.321(1.065, 1.721)	5.723(1.007–1.213)	0.015*
No	1	13	1	1	

*CI* Confidence interval, *COR* Crude odds ratio, *AOR* Adjusted odds ratio

*P < 0.05 = indicates statistically significant association

## Discussion

Proper use of EC within the right time interval would prevent unintended pregnancy and its damaging effects like unwanted childbirth and unsafe and risky abortion [21]. In our study majority of the students 279 (84.5%) heard about EC this is better compared to findings of a study conducted in Jimma (10.1%) [22], Fiche town (34.1%) [23], Mizan (73.3) [16] and Addis Ababa (84.2%) [23]. But relatively lower knowledge level in Mekelle (90.7%) [24] and Harar (93.5%) [18] This difference might be due better information education and communication (IEC) and media coverage in bigger cities regarding emergency contraceptives. Compared to study done abroad like that of Cameroon (63.0%) [25], and Nepal (63.7%) [26] The awareness level relatively higher this difference might be due to strong information education and communication activities done by Ethiopia.

In this study the main sources of EC information were peers and friends and health institutions unlike the findings of studies conducted in Harar and Addis Ababa where students got EC information from college (40.5%) and media (69.3%) respectively [20, 27]. Whereas the major source of source in studies conducted abroad such as Nepal (52.06%) [26] and Nigeria (31.8%) [28] Mentioned class room education and health works as their major sources of information.

Findings from this study concluded that little more than half (54.8%) of the study participants have a good knowledge about EC this is relatively higher compared to the findings of the study conducted in Arbaminch (21.9%) [9] and Haramaya (25.7%) [26], Mizan (34.6%) [16] (Nigeria (27.8%) [29]. However the knowledge level is lower in comparison to preparatory schools in Mekelle (75.7%) [22], Harar (70.0%) [20] and India (60.1%) [30]. this difference might be because of better access to medias and reproductive health related information in major cities.

According to findings of the current study out of 90 (27.2%) sexually active study participants 41% of them had a history of unprotected sex and only (40.5%) of them used EC afterwards. This utilization level better compared to findings from Addis Ababa (30.7%) [18], Harar (24.8%) [17], Mizan (31.7%) [16] and Nigeria from abroad 15.2% [31] from abroad. But lower compared to high schools in Mekelle (60.5%) [22] and 51.8% in Nepal [26].

The findings of this study showed that admission type (AOR = 7.421(1.241–4.041) *P* < 0.026), grade level of female students (AOR (4.21(3.451–4.172), *P* < 0.0140), (AOR 2.02 (1.641–9.071), *P* < 0.035) and discussion of reproductive health-related issues with parents (AOR 2.721 (0.231–2.612), *P* < 0.013) had a statistically significant association with knowledge of Emergency contraceptive. Students which are admitted to the night program are seven times more knowledgeable compared to those admitted to the regular program. This gap might be explained by the fact that students who attend night program are commonly more mature and old compared to regular students. Additionally, female students who are from senior grades were two times more knowledgeable of EC compared to junior year students. This finding is consistent with the finding of the study done in Harar [24] and Fiche town [23] the reason for this might be better exposure for emergency contraceptive related information as the students stay longer in the high schools.

According to findings from this study having a boyfriend showed a statistically significant association with the practice of EC (AOR 5.723 (1.007–1.213), *P* < 0.015), accordingly female students who have boyfriend are five times more likely to use EC than female students with no boyfriend. Furthermore, female students from senior classes have a better likelihood of using emergency contraceptive compared to junior class students, for instance female students from grade 12 are approximately three times more likely to use emergency contraceptives compared to grade nine students (AOR (2.83(0.231–1.549), *P* < 0.026).

## Conclusions

The above study showed an acceptable level of an overall EC knowledge factors such as admission type, grade level and discussion with parents about reproductive health issues were among the significant predictor of EC knowledge and senior students had better practice level of Emergency contraceptives To prevent unintended pregnancy among secondary school students, sexual and reproductive education focused on emergency contraceptives should be given promptly starting from an admission of the students to secondary school and parents should be encouraged to freely discuss reproductive health related matters with their children.

### Limitation

Since participation in the study was on voluntarily basis, the study might be affected by selection bias. Since the study design is cross-sectional it is difficult to declare causation.

## Data Availability

Additional materials will be available from the corresponding author on any reasonable request.

## Abbreviations

AOR: Adjusted odds ratio
CI: Confidence interval
COC: Combined oral contraceptive pill
COR: Crude odds ratio
EC: Emergency contraceptive
IUD: Intrauterine device
MTSS: Melke Tsedik Secondary School
POP: Progestin only pills
RZSS: Ras Zeselassie Secondary School
WSS: Wolkite Secondary School
YSPS: Yaberus Secondary and Preparatory School
